# Enhancing Virus Filter Performance Through Pretreatment by Membrane Adsorbers

**DOI:** 10.3390/membranes15010034

**Published:** 2025-01-17

**Authors:** Solomon Isu, Shu-Ting Chen, Raheleh Daneshpour, Hironobu Shirataki, Daniel Strauss, Andrew L. Zydney, Xianghong Qian, Sumith Ranil Wickramasinghe

**Affiliations:** 1 Ralph E. Martin Department of Chemical Engineering, University of Arkansas, Fayetteville, AR 72701, USA; solomon.isu@milliporesigma.com (S.I.); sc068@uark.edu (S.-T.C.); daneshpour@psu.edu (R.D.); 2 Scientific Affairs Group, Bioprocess Division, Asahi Kasei Medical, Chiyoda, Tokyo 100-0006, Japan; shirataki.hb@om.asahi-kasei.co.jp; 3 Research and Development, Asahi Kasei Bioprocess America, Glenview, IL 60026, USA; daniel.strauss@ak-bio.com; 4 Department of Chemical Engineering, The Pennsylvania State University, University Park, PA 16802, USA; zydney@engr.psu.edu; 5 Department of Biomedical Engineering, University of Arkansas, Fayetteville, AR 72701, USA

**Keywords:** aggregation, flux decline, fouling, membrane adsorber, monoclonal antibody, pH, prefiltration, reversible aggregate, virus filtration

## Abstract

Virus filtration is used to ensure the high level of virus clearance required in the manufacture of biopharmaceutical products such as monoclonal antibodies. Flux decline during virus filtration can occur due to the formation of reversible aggregates consisting of self-assembled monomeric monoclonal antibody molecules, particularly at high antibody concentrations. While size exclusion chromatography is generally unable to detect these reversible aggregates, dynamic light scattering may be used to determine their presence. Flux decline during virus filtration may be minimized by pretreating the feed using a membrane adsorber in order to disrupt the reversible aggregates that are present. The formation of reversible aggregates is highly dependent on the monoclonal antibody and the feed conditions. For the pH values investigated here, pretreatment of the feed using a hydrophobic interaction membrane adsorber was the most effective in minimizing flux decline during virus filtration. Ion exchange membranes may also be effective if the monoclonal antibody and membrane are oppositely charged. Consequently, the effectiveness of ion exchange membrane adsorbers is much more dependent on solution pH when compared to hydrophobic interaction membrane adsorbers. Size based prefiltration was found to be ineffective at disrupting these reversible aggregates. These results can help guide the development of more effective virus filtration processes for monoclonal antibody production.

## 1. Introduction

Virus filtration is routinely used in the manufacture of protein-based therapeutics such as monoclonal antibodies (mAbs). Validation of adequate virus clearance is critical in the manufacture of biopharmaceutical products. Virus filtration is a robust unit operation that makes use of membrane-based size exclusion to reject potentially contaminating virus particles. Virus filters are often characterized based on their rejection of model virus particles, typically minute virus of mice (MVM). The MVM capsid is about 25 nm in diameter and 2 nm thick and is arranged with a T = 1 icosahedral symmetry [1]. Virus filters are operated in normal flow mode under constant pressure. They are designed to provide at least 10,000-fold decrease in virus concentration in the permeate relative to the feed (4 log removal of virus, LRV). Additionally for the process to be economically viable, more than 95% product recovery is required. Design limits on product throughput (minimum product recovered per membrane surface area) and permeate flux put further constraints on the design of virus filters [2].

The size of antibodies in solution is highly variable and depends on buffer conditions. Roughly, biophysical analysis indicates they are around 12 nm long, with 3 rod-shaped arms each about 3.5 nm in diameter [3]. As membrane based size exclusion is usually most effective when the difference in size of the species to be separated is around an order of magnitude, the design criteria for virus filtration membranes is very demanding [4]. Since virus filtration occurs towards the end of the purification train, fouling of virus filters is most commonly due to product related contaminants such as product aggregates, dimers, trimers, and misfolded product molecules instead of host cell proteins [5].

Several recent studies have investigated an integrated ‘pretreatment’ step using either column chromatography or a virus prefilter to minimize fouling of the virus filtration membrane and improve the flux and throughput. These studies have indicated that the presence of product aggregates that are less than 100 nm in size are commonly responsible for the decrease in flux during virus filtration. While the feed stream is usually passed through a 0.2 or 0.1 μm sterilizing grade filter prior to introduction to the virus filter, smaller aggregates will not be removed and can then block the pores of the virus filter [6]. Further sized based prefiltration will not remove foulants that are similar in size to the product species. These foulants can potentially be removed by surface interactions. Brown et al. [7] used ion exchange membrane adsorbers to remove product related aggregates 8–13 nm in size. Rayfield et al. [8] indicated the importance of feed properties such as pH, buffer type, salt type, and concentration, all of which affect the biophysical properties of the mAb. This, in turn, can lead to greater aggregation and product related contaminants. Stanevich et al. [9] investigated the use of polyamide 6-6 (nylon 6-6) membranes as adsorptive prefilters, which function through hydrophobic interactions. Bolton et al. [10] used a prefilter that contained diatomaceous earth to bind aggregates through hydrophobic interactions. Others, such as Shirataki et al. [11], have used mixed mode resins as a pretreatment step prior to virus filtration.

Johnson et al. [12] provide a recent summary of current industrial practices in virus filtration, including the use of prefiltration. In another recent study, Isu et al. [13] describe the use of bioanalytical methods to identify aggregation-prone and less filterable proteoforms during virus filtration, which could guide the selection of an appropriate pretreatment strategy. Several investigators have noted that severe fouling is often observed even after prefiltration [5]. They note that this is due to adsorption of the mAb onto the virus filtration membrane surface. Furthermore, some investigators have noted severe membrane fouling even when the amount of high molecular weight species is less than 1% in the feed [8]. This is likely due to reversible self-association of the monomeric mAb [14].

Bieberbach et al. [15] indicate that these observations may be explained by considering the formation of irreversible and reversible aggregates [16,17,18]. Irreversible aggregates can be soluble or insoluble. They are held together by strong intermolecular forces such as covalent bonds. They can generally be detected by size exclusion chromatography. Reversible protein aggregation or self-assembly leads to the formation of soluble oligomers. They are held together by weak intermolecular interactions and are more likely to form at high concentrations that would exist in the narrow pores of a virus filter. These reversible aggregates may revert to protein monomers upon dilution [19]. Protein self-assembly is a concern in the manufacture and storage of biopharmaceutical products. Dynamic light scattering may be used to qualitatively determine the degree of protein self-assembly in solution [20].

The observations described above are further complicated by the fact that virus filters from different manufacturers are made of different polymeric materials [8]. Proprietary surface treatments are used to make the membrane surface more biocompatible and to suppress fouling. However, the degree of adsorption of a specific mAb, including both self-assembled mAb species as well as irreversible aggregates, will depend on the mAb, the specific surface properties of the membrane, and the buffer conditions.

In this study, we have investigated the performance of the Planova BioEx virus removal filter (Asahi Kasei Bioprocess, Glenview, IL, USA) using amAb under different pH conditions. Size based membranes, as well as ion exchange and hydrophobic interaction membrane adsorbers, have been used as a prefiltration/pretreatment step. The impact of these pretreatment steps on the permeate flux through the virus filter has been determined. Dynamic light scattering has been used to determine the level of self-assembly of the mAb under different pH conditions. Three pH conditions were considered: above (pH 8.6), around (pH 7.5), and below (pH 5.0) the isoelectric point of the mAb. Due to the stability of the mAb, pH values much above the isoelectric point of the mAb could not be investigated. While the pH values investigated are of relevance industrially, they also enable investigation of the behavior of the mAb at pH values below, near, and above its isoelectric point. The results provide guidelines for the selection of a prefilter/membrane adsorber to be used prior to virus filtration.

## 2. Materials and Methods

### 2.1. Reagents and Filters

The reagents used for preparing the various buffers were sodium acetate trihydrate (molecular biology grade, >99% purity), sodium chloride, sodium phosphate monobasic monohydrate (ACS reagent, >98% purity), sodium phosphate dibasic (reagent plus, >99% purity), and glacial acetic acid from MilliporeSigma (Billerica, MA, USA). Ammonium sulfate was obtained from VWR Life Science (Radnor, PA, USA). Ultrapure water with a resistivity of 18.2 MΩ was used for buffer formulation.

The mAb used in these studies had a molecular weight of 148 kDa and an isoelectric point of 8.0. Four different feed buffer solutions were prepared consisting of 20 mM sodium acetate at pH values of 5.0, 7.5, and 8.6. The pH was adjusted using glacial acetic acid. At pH 5.0 and 8.6, 200 mM NaCl was added. At pH 7.5 the buffer was prepared with and without 200 mM NaCl. The mAb concentration in all solutions was 5 g L^−1^.

The prefilters and membrane adsorbers used in this study are summarized in Table 1. In this work, the prefilter or membrane adsorber was not installed in-line with the virus filter. Instead, the pretreated product solution was introduced to the virus filter within 5 min of prefiltration. The virus filter used was the Planova BioEx with membrane surface area 0.0003 m^2^ (Asahi Kasei Bioprocess, Glenview IL, USA). All feed streams were prefiltered through a 0.2 μm bottle top filter. Results for the 0.2 μm bottle top filter did not include any further pretreatment. Results for all other prefilters and membrane adsorbers were generated for feed streams after passage through the 0.2 μm bottle top filter. The feed from the 0.2 μm bottle top filter was used within 5 min either as the feed to the virus filter or the feed to the subsequent pretreatment step.

Membrane adsorbers were installed on a fast protein liquid chromatography (FPLC) system (Cytiva, Marlborough, MA, USA). For hydrophobic interaction chromatography (HIC) membrane adsorber pretreatment (at all pH values 5.0, 7.5, 8.6), the buffer included 200 mM NaCl. For ion exchange membrane adsorbers, 200 mM NaCl was present in the buffer at pH 5.0 and 8.6 only. For the sized based prefilters (0.1 μm bottle top filter and 75 N,) no NaCl was added to the feed.

### 2.2. Analytical Methods

Buffer pH and conductivity were measured using an Orion Star™ pH/conductivity benchtop multiparameter meter from ThermoFisher Scientific (Waltham, MA, USA). The mAb concentration was measured by UV absorbance at 280 nm using a Genesys10 UV Scanning System (Waltham, MA, USA) with VWR Quartz Spectrophotometer Cell (path length 1 cm). Dynamic light scattering (DLS) was performed using a Delsa™ Nano particle size analyzer from Beckman Coulter (Brea, CA, USA) to determine the hydrodynamic diameter of the mAb. Results represent the average of three readings.

Size exclusion chromatography (SEC) was conducted to determine the aggregate fraction in the mAb solution at pH 5.0 after prefiltration through the 0.2 μm bottle top filter, after filtration through the BioEx, and in the buffer flush performed after mAb filtration. A TSKgel G3000SWXL column with 7.8 mm ID, 30 cm length, and particle size of 5 μm (Tosoh Bioscience, Grove City, OH, USA) was used. The column was installed on a high-performance liquid chromatography (HPLC) instrument, Agilent 1260 Infinity Quaternary LC, (Agilent Technologies, Santa Clara, CA, USA). The mobile phase was 20 mM sodium phosphate buffer, pH 7.0, with 300 mM ammonium sulfate. The SEC column was equilibrated with the mobile phase at 0.9 mL min^−1^ for one hour before sample introduction. All samples were filtered through a 0.2-μm syringe filter and loaded into 1 mL sample vials. The HPLC run cycle was twenty minutes at a flow rate of 0.9 mL min^−1^. Subsequently, 10 μL samples were injected into the column and analyzed.

### 2.3. Virus Filtration

During virus filtration, the weight of the permeate was recorded. Visual leak integrity tests (as described by the manufacturer) were performed on the BioEX virus filter at 100 kPa for 20 s before flushing air out of the system. Deionized water filtered with a 0.2 μm bottle top filter was added into the Planova™ Pressure Reservoir (Asahi Kasei Bioprocess, Glenview, IL, USA). The virus filter was flushed with 40 L m^−2^ of DI water and then with 40 L m^−2^ of the feed buffer. The mAb loading volume was about 65 mL (250 L m^−2^) at a feed pressure of 310 kPa. For feed streams prefiltered with the 0.2 μm bottle top filter only, a buffer flush was included.

## 3. Results

In this study, 200 mM NaCl was not added to all the feed streams at pH 7.5, which was close to the isoelectric point of the protein. This was performed to minimize aggregate formation [21,22]. When the pH of the solution is close to the isoelectric point of the mAb, a high ionic strength buffer can cause ‘salting-out’, which could lead to increased aggregate formation. However, 200 mM NaCl was added to all feed streams that were pretreated with the HIC membrane adsorber as adsorption of the impurities onto the hydrophobic ligands is enhanced at high salt concentrations.

Dynamic light scattering was used to determine the diameter of the mAb for all feed streams after prefiltration. Table 2 summarizes the results. As can be seen, there is considerable variability in the diameter of the mAb. Without additional pretreatment beyond the 0.2 μm prefilter, the average diameter was largest for the mAb at pH 5.0. The hydrodynamic diameter was lowest close to the isoelectric point of the mAb, even though these conditions would be expected to minimize electrostatic repulsive interactions. Interestingly, the diameter was larger for the pH 5.0 feed prefiltered through the Planova 75 N than that filtered through the 0.1 and 0.2 µm prefilters, even though the 75 N has a significantly smaller pore size.

**Figure 1:**
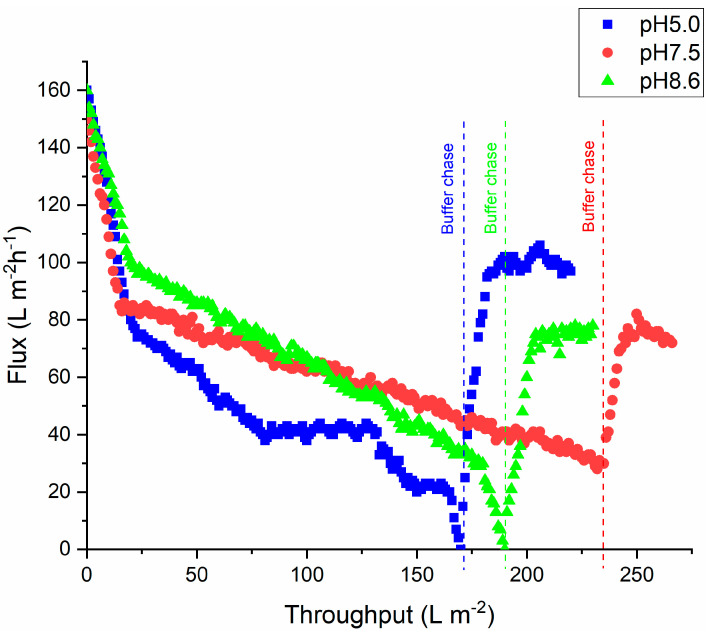
Variation of virus filter flux versus throughput for feed streams at different pH after pretreatment with a 0.2 μm bottle top filter. When flux values dropped to below 40 L m^−2^ h^−1^, a buffer flush was initiated and the flux increased.

Figure 1 gives the virus filter flux as a function of throughput for the three pH values tested for the feed stream pretreated only using a 0.2 μm bottle top filter. Though the aim was to load the virus filter to 250 L m^−2^, due to rapid flux decline this was not achieved. The filtration was stopped when the flux dropped below 40 L m^−2^ h^−1^, at which point the buffer flush using 20 mL of the feed buffer was commenced. As can be seen, the flux increases somewhat during the buffer flush. The flux decline is most rapid at pH 5.0, as is the flux recovery during the buffer flush. The flux decline appears to be least at pH 7.5, which is close to the isoelectric point of the mAb, with the flux remaining >40 L m^−2^ h^−1^ out to a throughput of 230 L m^−2^.

Since the feed stream at pH 5.0 displayed the most rapid flux decline, samples of the feed and permeate after the virus filtration and after the buffer flush were analyzed by SEC. The results are shown in Table 3. It can be seen that the percentage of dimer is very low in all samples. No higher molecular weight oligomers were detected.

**Figure 2:**
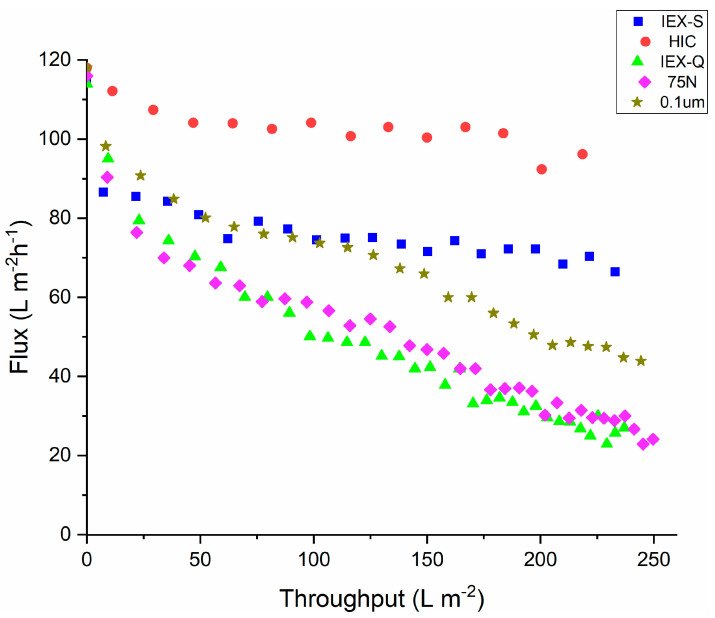
Variation of permeate flux through the BioEx virus filter versus throughput for feed streams pretreated using the various prefilters and membrane adsorbers listed in Table 1. The feed pH was 5.0.

Figure 2 gives the permeate flux through the Planova BioEx virus filter as a function of throughput at pH 5.0 for feed streams pretreated using the various prefilters and membrane adsorbers listed in Table 1. All feed streams were passed through the 0.2 μm bottle top filter before further pretreatment. Results for pretreatment with the 0.2 μm bottle top filter only are given in Figure 1. As can be seen for all the different pretreatment methods, the flux through the virus filter decreases with time. The hydrophobic interaction membrane adsorbers performed the best as the decrease in flux is least for feed streams pretreated with this membrane adsorber, with the flux remaining above 95 L m^−2^ h^−1^ out to a throughput of 250 L m^−2^. The next best performance was obtained after pretreatment with the cation exchange adsorber (IEX-S).

**Figure 3:**
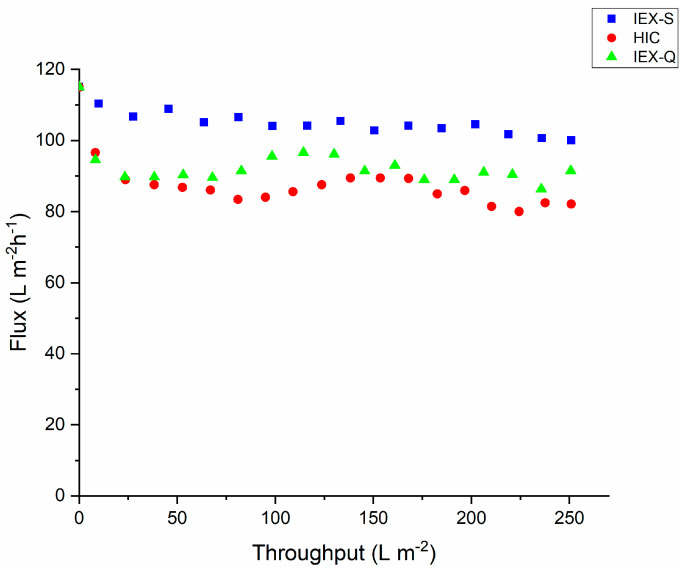
Variation of permeate flux through the BioEx virus filter versus throughput for feed streams pretreated using the various membrane adsorbers listed in Table 1. The feed pH was 7.5.

Figure 3 shows analogous results to Figure 2 but for a feed pH of 7.5. While pretreatment using the IEX-S gave the highest flux, all three membrane adsorbers provided a stable flux over 250 L m^−2^ throughput. Note that the permeate flux through the feed pretreated with only the 0.2 µm bottle top filter (without any membrane adsorber) declines to <40 L m^−2^ h^−1^ after 250 L m^−2^ throughput under otherwise identical conditions.

**Figure 4:**
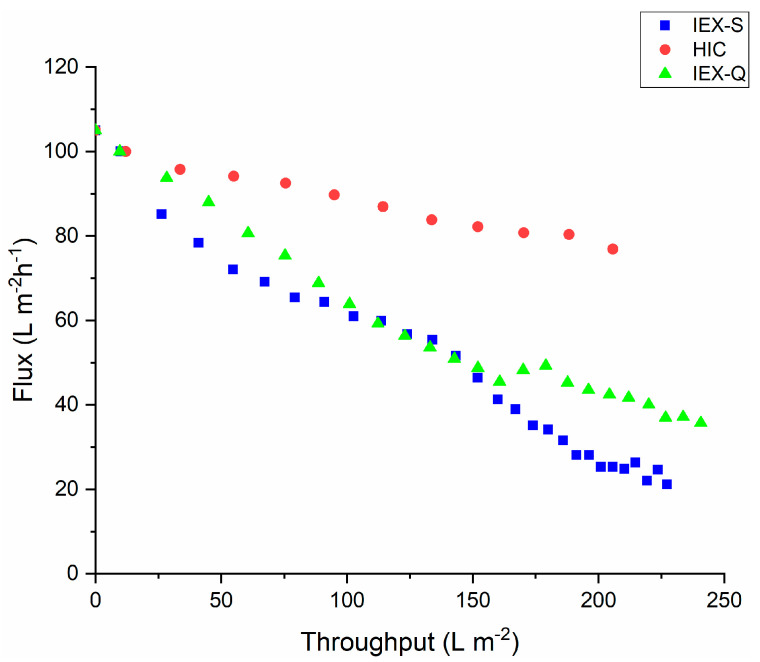
Variation of permeate flux through the BioEx virus filter versus throughput for feed streams pretreated using the various membrane adsorbers listed in Table 1. The feed pH was 8.6.

Finally, results for permeate flux through the BioEx filter for feed streams at pH 8.6 prefiltered using the three membrane adsorbers are given in Figure 4. As can be seen, a significant flux decay was observed throughout the run for all three conditions, with the least flux decline obtained using the HIC membrane adsorber. The results with the IEX-S adsorber were similar to those obtained in Figure 2 for the bottle top filter alone, suggesting that the negatively-charged adsorber is unable to remove any significant aggregates when the pH of the solution is above the pI of the antibody, i.e., under conditions where the antibody is negatively-charged.

## 4. Discussion

The results in Figure 1 indicate that at pH values below, around, and above the isoelectric point of the mAb, rapid flux decline is observed during virus filtration when the feed stream is only prefiltered through a 0.2 μm filter. The SEC data (Table 3) for the feed stream at pH 5.0, which gave the most rapid flux decline during virus filtration, indicate the absence of any higher molecular weight oligomers while the percentage dimer is only around 5%. On the other hand, the DLS data (Table 2) indicate that the hydrodynamic diameter is the largest for the feed stream at pH 5.0 with an average size of 21.4 nm, which is essentially the same as the mean pore diameter of the BioEx virus removal filter. These data suggest that fouling is due to reversible aggregate formation, with these aggregates detected by DLS but not by SEC due to dilution of the protein feed by the buffer used in the SEC analysis. Further evidence of this is provided by the fact that there is rapid flux recovery during the buffer flush under all conditions, something that would not be expected if the fouling were due to irreversible aggregates that block the pores. Note that Billups et al. [2] also observed rapid flux recovery during the buffer flush due to dilution of the mAb and the dissociation of these reversible aggregates. The flux decline is intermediate at pH 8.6 and least at pH 7.5. The degree of flux decline is correlated to the measured hydrodynamic diameter (Table 2), with the smallest hydrodynamic diameter of 11.3 nm observed at pH 7.5.

Figure 2 indicates that rapid flux decline is observed at pH 5.0 for the feed streams that were prefiltered with just one of the size based prefilters. Interestingly, the flux decline is less pronounced for the feed processed through the 0.1 μm pore size prefilter than the 75 N even though the 75 N has a smaller nominal pore size. This difference may be associated with the different chemistry of these prefilters (Table 1); the 0.1 µm prefilter is a polyethersulfone membrane while the 75 N is regenerated cellulose. The more hydrophobic polyethersulfone membrane may provide better removal of foulants by adsorptive interactions, consistent with the strong performance of the HIC membrane adsorber in arresting the flux decline during virus filtration. Thus, it appears that membrane hydrophobicity plays an important role in disrupting or removing the reversible aggregates that form at pH 5.0.

The IEX-Q and IEX-S membranes are cellulosic with quaternary amine and sulfonic acid ligands, respectively. At pH 5.0, the mAb will be positively charged. Thus, there will be a net repulsion from the quaternary amine ligands on the IEX-Q membrane while there will be a strong attractive interaction with the sulfonic acid ligands on the IEX-S membrane. This attractive interaction provides more effective disruption/removal of the reversible aggregates, leading to the greater effectiveness of the IEX-S membrane adsorber at arresting the flux decline during virus filtration.

The hydrodynamic diameter after pretreatment provides a qualitative indication of the likelihood of rapid flux decline during virus filtration. The feed stream after pretreatment with the HIC membrane adsorber had the smallest hydrodynamic diameter and, in turn, the highest and most stable flux during virus filtration.

The variation of virus filter flux at pH 7.5, which is close to the isoelectric point of the mAb, is very different than that observed at pH 5.0. Based on the results in Figure 2 and Figure 3, it appeared that sized based prefiltration is not effective at arresting the decline in flux. Consequently, only the membrane adsorbers were investigated. All three adsorbers gave similar results, with relatively stable flux, consistent with the similar hydrodynamic diameter for these three cases (Table 2). Note that both the hydrodynamic diameter and fouling behavior were similar to that observed for the feed stream at pH 5.0, which was pretreated with the HIC membrane adsorber.

Protein aggregation (reversible and irreversible) is complex and highly dependent on the specific mAb. Generally, aggregation implies the formation of non-native oligomeric species by interactions between ‘modified’ monomers [23]. Reversible aggregation, however, refers to the noncovalent interactions between native monomers. Interactions between mAb monomers can be induced because of the charge distribution, charge heterogeneity, and surface hydrophobicity [24]. Esfandiary et al. [25] indicated that reversible aggregation or self-association consists of both enthalpic and entropic contributions. Electrostatic interactions, however, are more enthalpic in nature while hydrophobic interactions are more entropic in nature [26]. The electrostatic effect is generally more dominant. This would explain the much smaller hydrodynamic diameter when the solution pH is close to the mAb isoelectric point.

When the pH of the solution is close to the isoelectric point of the mAb, the net charge of the mAb will be close to zero. Thus, hydrophobic patches, as well as positive and negative charge patches, will be able to interact with the HIC, IEX-S, and IEX-Q membranes. It is also important to note that at pH 7.5, no NaCl was added to the feed nor to the IEX-S and IEX-Q membranes. The charge screening effect of higher ionic strength buffers will be absent, thus increasing possible interactions between the IEX ligands and the mAb. It appears that interactions between the membrane and mAb are important to suppress, at least temporarily, the reversible mAb aggregation. In the case of the 0.2 μm prefilter, the hydrophobic interactions will be much weaker as the feed buffer did not contain 200 mM salt, perhaps explaining the observed decline in permeate flux through the BioEx virus filter.

The observed decreases in permeate flux at pH 8.6, shown in Figure 4, are in general agreement with the above discussion. The hydrodynamic diameter of the mAb after pretreatment with the three membrane adsorbers is smallest for the HIC and IEX-Q membranes. It is largest for prefiltration with the 0.2 μm filter, which also shows the greatest flux decline during virus filtration (Figure 1). Although pretreatment with the HIC membrane adsorber leads to the highest permeate flux, a noticeable flux decline is observed compared to feed streams at pH 5.0 and 7.5. Furthermore, the hydrodynamic radius is larger at pH 8.6 than at pH 5.0 or 7.5. Reversible and irreversible mAb aggregation is highly dependent on solution conditions. Thus, it is not surprising that the hydrodynamic diameter after HIC pretreatment is larger at pH 8.6 compared to the lower pH values. At pH values greater than the isoelectric point of the mAb, the mAb will be negatively charged. Thus, it will interact more strongly with the quaternary ammonium ligands of the IEX-Q membrane adsorber. Table 2 indicates that the hydrodynamic diameter of the mAb is smaller after pretreatment with the IEX-Q membrane adsorber compared to the IEX-S membrane adsorber, although the flux decline is only slightly improved for the feed stream pretreated with the IEX-Q membrane adsorber compared to the IEX-S membrane adsorber.

**Figure 5:**
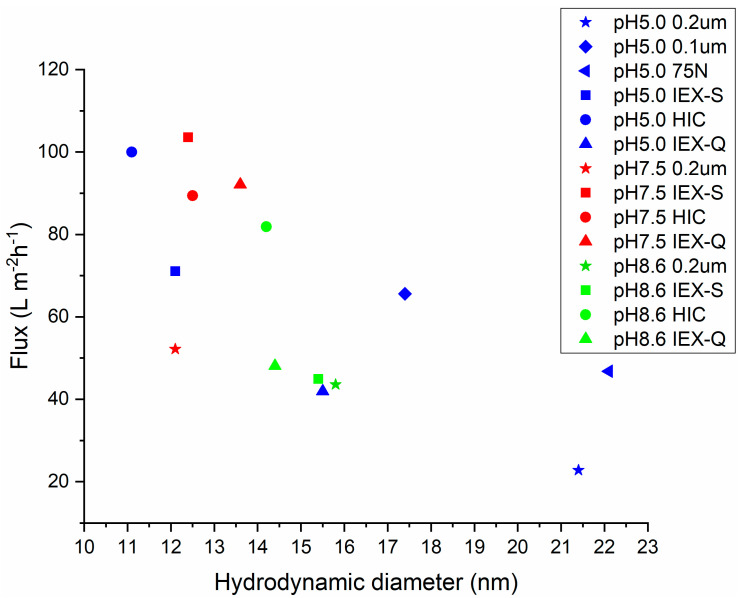
Variation of permeate flux through the BioEx virus filter versus hydrodynamic diameter using the various pretreatments listed in Table 2. Results are given for virus filter flux at 150 L m^−2^ throughput.

Our results indicate that determining the hydrodynamic diameter of the mAb by DLS provides a qualitative indication of the likely flux decline. Figure 5 gives the variation of virus filter flux at 150 L m^−2^ with mAb hydrodynamic diameter for the various experiments conducted here. As can be seen, though, there is some scatter; a lower hydrodynamic diameter is a good indication of the absence of reversible aggregates and higher virus filter flux.

Taken together, our results indicate that pretreatment with the HIC membrane adsorber was generally the most effective in arresting the flux decline observed during virus filtration. Billups et al. [2] also noted that hydrophobic interactions between the mAb and a membrane placed directly on top and upstream of the virus filtration membrane was successful in disrupting reversible aggregates. Our results indicate that electrostatic interactions between the mAb and membrane can also lead to dissociation of reversible aggregates and an improvement in virus filter flux. However, the effectiveness of the ion exchange membrane adsorbers is much more pH sensitive, with significant reductions in flux decline only observed when the mAb and ion exchange ligands are oppositely charged. A similar result was observed by Shirataki et al. [11] using mixed mode resins. Since the formation of reversible aggregates is highly dependent on solution conditions, the pH and ionic strength could potentially be adjusted to minimize the formation of reversible aggregates.

In addition, the formation of reversible aggregates is likely to be highly time dependent. Once disrupted, the reversible aggregates can reform with time. Consequently, using an in-line pretreatment step is likely the most effective approach to reduce fouling. It is possible that an in-line pretreatment step would be more effective than the separate pretreatment and virus filtration steps conducted here [7]. Further, while DLS measurements provide an indication of the degree of self-association of the mAb in solution, the environment in the constricted pores of a virus filter is very different and may well promote the formation of reversible aggregates due to the increase in local mAb concentration.

This investigation was conducted using only one mAb and at one mAb concentration, 5 g L^−1^. While this value is representative of mAb concentrations used industrially, the formation of reversible aggregates is highly concentration dependent. In fact, as shown here, during the buffer chase due to dilution of the mAb, the reversible aggregates present are disrupted, leading to higher permeate fluxes during virus filtration. Future work will focus on the effect of mAb concentration on the disruption of the reversible aggregate and the effectiveness of pretreatment.

For ion exchange membrane adsorbers, charged patches on the aggregate surface interact with the oppositely charged ligands on the membrane surface. For hydrophobic interaction membrane adsorbers, hydrophobic patches on the aggregate surface interact with hydrophobic ligands present on the membrane surface. It is likely that if the ligands on the membrane adsorber interact with the reversible aggregates, it will lead to disruption of the reversible aggregates rather than adsorption given the weak interactions between mAb molecules. Further investigation is needed to determine the extent of reversible aggregate disruption versus adsorption.

It should be noted that besides the formation of reversible aggregates, flux decline during virus filtration after the feed has been prefiltered through a 0.2 or 0.1 μm filter can occur due to other product-related contaminants. Insoluble dimers formed from non-native mAb species can easily pass through a 0.2 or 0.1 μm filter. These dimers should be detectable by SEC. Since denatured mAb monomers are more hydrophobic than the native mAb monomers, the use of a HIC membrane adsorber may be effective. Jones et al. [27] indicate that certain ‘high-risk’ host cell proteins can also associate with the mAb product and thus pass through the purification train and copurify with the mAb. While these species can easily pass through the pores of a 0.2 or 0.1 μm prefilter, they can lead to fouling of the virus filter. These authors indicate various analytical methods such as mass spectrometry and HCP ELISA assays may be useful to detect the presence of these high-risk HCPs.

## 5. Conclusions

As virus filtration occurs towards the end of the purification train, fouling of virus filters is usually due to product related contaminants, including the presence of reversible aggregates. These aggregates can easily pass through a 0.2 or 0.1 μm prefilter, making these size-based prefilters largely ineffective in protecting the virus removal filter. Insoluble dimeric aggregates can form from non-native product molecules. These aggregates can be detected by SEC. However reversible aggregates can form from the native mAb. The formation of these reversible aggregates is concentration dependent; dilution of the feed will cause them to revert to the monomeric state; thus, they cannot be detected by SEC. However, DLS measurements can provide at least some indication of the presence of these reversible aggregates.

Membrane adsorbers can be used to disrupt the reversible aggregates that form and hence arrest the flux decline during virus removal filtration. It is important that the reversible aggregates interact with the ligands present in the membrane adsorber. HIC membrane adsorbers were found to be an effective pretreatment step over a range of feed pH values. Ion exchange membrane adsorbers can also reduce the fouling during virus filtration, but their effectiveness is much more pH dependent. For ion exchange adsorbers, significant improvement in virus filter performance was only obtained when the mAb and adsorber ligands are oppositely charged.

## Figures and Tables

**Table 1:** Details of prefilters and membrane adsorbers used in this study.

Prefilter	Membrane Material	Source	Feed Streams Tested	Mechanism of Action	Comment
0.2 μm bottle top filter	Polyethersulfone	ThermoFisher Scientific (Waltham, MA, USA)	Acetate buffer with 200 mM NaCl at pH 5.0, 7.5 and 8.6. Acetate buffer with no added NaCl at pH 7.5.	Size exclusion	Used for all feed streams. Tested by itself for feed stream at pH 5.0 and 8.6 with 200 mM NaCl; pH 7.5 without 200 mM NaCl.
0.1 μm bottle top filter	Polyethersulfone	ThermoFisher Scientific (Waltham, MA, USA)	Acetate buffer with 200 mM NaCl at pH 5.0.	Size exclusion	Filtered through 0.2 μm bottle top filter, then through 0.1 μm bottle top filter.
Planova 75 N (75 N)	Regenerated cellulose hollow fibers, 0.001 m^2^ surface area, nominal pore size 75 nm	Asahi Kasei Bioprocess (Glenview, IL, USA)	Acetate buffer with 200 mM NaCl at pH 5.0.	Size exclusion	Filtered through 0.2 μm bottle top filter, then through Planova 75 N filter.
Sartobind Q (nano) (IEX-Q)	Cellulosic membrane, quaternary ammonium ligands, 3 mL bed volume, 8 mm bed height, pore size > 3 µm.	Sartorius AG, (Göttingen, Germany)	Acetate buffer with 200 mM NaCl at pH 5.0 and 8.6. Acetate buffer with no added NaCl at pH 7.5.	Anion exchange	Filtered through 0.2 μm bottle top filter, then through Sartobind Q adsorber.
Sartobind S (nano) (IEX-S)	Cellulosic membrane, sulfonic ligands, 3 mL bed volume, 8 mm bed height, pore size > 3 µm.	Sartorius AG, (Göttingen, Germany)	Acetate buffer with 200 mM NaCl at pH 5.0 and pH 8.6. Acetate buffer with no added NaCl at pH 7.5.	Cation exchange	Filtered through 0.2 μm bottle top filter, then through Sartobind S adsorber.
Sartobind Phenyl (HIC)	Cellulosic membrane, phenyl ligands, 3 mL bed volume, 8 mm bed height, pore size > 3 µm.	Sartorius AG, (Göttingen, Germany)	Acetate buffer with 200 mM NaCl at pH 5.0, 7.5, 8.6.	Hydrophobic interaction	Filtered through 0.2 μm bottle top filter, then through Sartobind Phenyl adsorber.

**Table 2:** Average mAb diameter measured by dynamic light scattering for the various feed streams after pretreatment.

Pre-Filter	Average Diameter in Permeate (nm)	Buffer Condition
IEX-S	12.1	pH 5.0
HIC	11.1	
IEX-Q	15.5	
0.1 μm	17.4	
0.2 μm	21.4	
75 N	22.1	
IEX-S	12.4	pH 7.5
HIC	12.5	
IEX-Q	13.6	
0.2 μm	12.1	
IEX-S	15.4	pH 8.6
HIC	14.2	
IEX-Q	14.4	
0.2 μm	15.8	

**Table 3:** SEC data for the feed and permeate after virus filtration and buffer flush. The feed pH was 5.0.

	Feed	Permeate: Virus Filtration	Permeate: Buffer Flush
Oligomer	0%	0%	0.5%
Dimer	5.2%	0%	1.4%
Monomer	94.8%	100%	98.1%

## Data Availability

The original contributions presented in this study are included in the article. Further inquiries can be directed to the corresponding authors.
